# General cognitive status among Baby boomers and pre-boomers in Taiwan: the interplay between mid-life socioeconomic status and city residence

**DOI:** 10.1186/s12877-017-0503-7

**Published:** 2017-05-25

**Authors:** Chi Chiao

**Affiliations:** Insitute of Health and Welfare Policy, Research Center for Health and Welfare Policy, College of Medicine, National Yang-Ming University, Taipei, Taiwan, Republic of China

**Keywords:** General cognitive status, Mid-life SES, Baby boomers, City residence, Taiwan

## Abstract

**Background:**

This study seeks to assess the interaction between mid-life socioeconomic status (SES) and city residence on the cognitive status of Baby Boomers and pre-Boomers in Taiwan, a non-Western society with a distinct cultural and family context, taking apolipoprotein E (APOE) gene polymophism and life stressors into consideration.

**Methods:**

The data used was from the Social Environment and Biomarkers of Aging Study (SEBAS) collected in Taiwan during 2006, this involved 1245 individuals from 23 communities and used multilevel regression. General cognitive status was assessed by ten questions via personal interviews. The questions were part of the Short Portable Mental Status Questionnaire, a 10-item free-recall and immediate recall test. Mid-life SES was defined by education and major mid-life occupation of the participant and/or their partner.

**Results:**

Mid-life SES was positively associated with cognitive status among both Baby Boomers and pre-Boomers, even after adjusting for APOE polymorphism and stressor covariates. For Baby Boomers, city residents were more likely than town residents to show better cognition (β = 1.47, *p* < 0.01) and an interaction effect between mid-life SES and city residence was observed (β = −2.12, *p* < 0.01). While both the Baby Boomer and pre-Boomer cohorts who lived with a partner were reported better cognition, the effects of depressive symptoms and ethnicity differed by cohort.

**Conclusions:**

Having a high level of mid-life SES and living with a partner are associated with better cognition for both cohort groups. An interplay effect between mid-life SES and place of residence on cognition was only found for Baby Boomers. On the other hand, being psychologically depressed was associated with poorer cognition among pre-Boomers. These results underscore the specific roles of mid-life SES, city residence, and life stressors with regard to the cognitive status of Baby Boomers and pre-Boomers in Taiwan.

## Background

Cognitive impairment of older adults has often been associated with subsequent dementia; this then contributes to the global burden associated with this disease, as well as affecting the quality of life of both individuals and families. Given this reality, there has been significant scholarly interest in understanding the relationship between severe cognitive impairment and various genetic factors. Such relationships have been well-documented in neuropsychiatric studies [1] and a gene-environment interaction hypothesis has been proposed with respect to the apolipoprotein E4 (APOE4) allele. This allele has a strong association with presence of various forms of severe cognitive impairment, including Alzheimer’s disease [2, 3]. Environment variables have also brought forward as possible pathways that may influence cognitive status [4, 5].

A related, but somewhat separate hypothesis, which is based on the exposure-disease-stress model, provides a framework that underscores the importance of the inclusion of social stress as a major environmental component in this type of study [6]. This model hypothesizes the spatial boundaries associated with the residence of various socioeconomic groups has an effect. This model also pays particular attention to individuals in the context of their residential place in relation to their health status and therefore includes various biological, psychosocial, and socioeconomic components, as well as considering the extent and intensity of the associational links. Accordingly, the present study hypothesizes that individual socioeconomic status (SES) and the residential context of individuals, together with related stressor factors, work together to create a life stress level that serves as a foundation and exerts a negative influence on an elderly individual’s health, including their cognitive status.

Although recent studies have suggested that disadvantages experienced in early life of an individual may affect the brain [7] and cognitive development [8, 9], few investigations have addressed the effects of such disadvantages during a particular cohort’s life, particularly in relation to the individual’s family and even their residential context. Baby Boomers are a particularly interesting social group to investigate regarding this question as it is this generation of individuals, born between 1946 and 1964, who have experienced the rapid global revival after World War II. Given their higher levels of education, and as a consequence their greater employment in professional and managerial positions, the socioeconomic context of these Baby Boomers is markedly different from that of members of the preceding generation [10–12].

East Asian countries such as Taiwan have a historical background that includes a Confucian ideology and this common tradition emphasizes filial piety in the family context; this acts as an important cultural force that shapes individual economic and family roles [13–15]. The present study thus hypothesizes mid-life SES served as a fundamental factor that affects the cognitive health outcomes of older adults in their family spheres [1, 8, 14]. Beginning in approximately the 1960s, the positive influence of an expansion in exports on economic growth has been recognized for four East Asian countries, including Taiwan, which were named the “Asian Tigers” or “Asian Dragons” [16–18]. Given both an improved access to education and the rapid level of economic growth and industrialization in these countries, the Baby Boomer cohort was more economically active mid-career than their pre-Boomer counterparts. As a result, the Baby Boomers are more likely to have struggled with a conflict between work achievement and their caregiving role in the family [10, 11]. Thus they have been the sandwich generation [15]. In addition, there was a major growth in the number of city dwellers during this period because of the increased employment opportunities in the cities over the same period [15]. The present study builds upon the exposure-disease-stress framework [6] and further hypothesizes that place of residence is another contributing factor that may explain variations in cognitive status when assessing the influence of mid-life SES. Moreover, a question that still remains unanswered is whether the association between mid-life SES and cognitive status is stronger for all Baby Boomers, notwithstanding their cognitive status, who lived through a highly changing social context, namely in a city area after the World War II, or whether those Baby Boomers with a high mid-life SES benefited more from having a residence in a city.

The exposure-disease-stress framework suggests that mid-life SES disadvantaged groups disproportionately experience a range of adversities and as a result such groups are more likely than others to exhibit poor health [6]. Perceived stressors are often active throughout their lives within their specific family context. In this regard, economic strain due to financial difficulties is considered to be one of the more important sources of life stress and this has been found to be related to, but is independent of, mid-life SES [19, 20]. Research has provided little evidence supporting the effects of family stress in this context, but researchers have argued that stressors resulting from family living arrangements and relationships within the family context may manifest themselves as an association between SES and health outcomes [9, 21, 22]. On the other hand, a series of studies has provided evidence that taking part in physical activity appears to be a buffer the relationship between SES and cognitive status [23, 24].

As a result of the knowledge gaps associated with prior research, investigations of SES effects have relatively less often used the social causation hypothesis that highlights mid-life SES as an important antecedent to an increased likelihood of cognitive problems in later life. Furthermore, there has also been little research on the effect of these factors on Baby Boomers in a non-Western society. Based on the above, a further examination of this topic specifically targeting Taiwan as a non-Western society in particular appears to be warranted. The present study used data from a population-based survey of older adults in Taiwan and explores whether mid-life SES and city residence are jointly and significantly associated with differences in late-life cognitive status among a Baby Boomer cohort and among a pre-Boomer cohort. This analysis specifically takes into account APOE polymorphism as one covariate in the investigation. Furthermore, an additional gap in the literature was also addressed, namely whether or not life-related stressors and buffers are associated with the relationship between mid-life SES, city residence, and cognitive status.

## Methods

### Study population

The study participants were adults aged 53 or older who formed the 2006 Social Environment and Biomarkers of Aging Study (SEBAS), which is a subsample of the respondents from the Taiwan Longitudinal Survey of Ageing (TLSA). The SEBAS started in 2000; however, a Baby Boomer cohort of those aged 53–60 in 2006 was not added until 2006. The inclusion of this refresher cohort was to ensure that the SEBAS 2006 comprised a representative sample of the Taiwanese population aged 53 and older. In order to assess the research goal of this study, the analysis has been restricted to the 2006 SEBAS, which includes both pre-Boomers and Baby Boomers. The 2006 SEBAS sample was randomly selected from the 1999 and 2003 waves of the TLSA. It oversampled urban residents living within 23 community (township) clusters. Further details on the SEBAS sampling and design are reported elsewhere [25]. This study focused on the general cognitive status of Baby Boomers and pre-Boomers. The analytical sample is further restricted to the 2006 SEBAS members who self-reported on cognition and therefore the final sample consists of a total of 1245 individuals. The study protocol was approved by the Ethical Committee of National Yang-Ming University.

### Measures

General cognitive performance was assessed by ten questions posed during the personal interviews. The questions were part of the Short Portable Mental Status Questionnaire (SPMSQ) [26], a 10-item free-recall test [27], and of a modified Digits Backward test [28]. Among the ten questions, the eight from the SPMSQ were: ‘what are the day, the month, and the year’; ‘what day of the week is it’; ‘how old are you’; ‘what is your home address’ and ‘count backwards from 20 by 3 a total of four times’. This part of the measure is based on the correct answer count and has a possible range of 0 to 13. The 10-item free-recall test was to recall 10 words and this has a possible range of 0 to10. One question from the modified Digits Backward test was to reverse the order of the numbers that the interviewers had read out to respondents. The latter two questions assessed the participants’ memory span, which has been shown to be associated with the risk of developing dementia [29]. The total score for cognitive performance thus ranged from 0 to 24 and a higher score was indicative of a better cognitive status (Cronbach’s α = 0.71). Use of these ten questions as cognitive tests has been validated using the Chinese equivalent of the Mini-Mental State Examination (MMSE) [30, 31].

The primary variables of interest are mid-life SES and city residence. Mid-life SES consisted of self-reported measures related to the subject’s and spouse’s education and the primary lifetime occupation of the participants and their spouses. The highest education attainment of the participants and their spouses was categorized into either low (less than elementary school) or high (completed elementary school or higher) [32]. The highest occupation of participants and their spouses was categorized into either low (manual, unemployed or housekeeper) or high (non-manual occupation) [33]. As was carried out in a prior study [8], the education and occupation measures were added together to produce a composite index that had a range from 1 to 3. Participants high on both (a high family SES) were coded as 3, while participants low for one or other (a medium family SES) were coded as 2, and participants low for both (a low family SES) were coded as 1.

City residence was another focal explanatory variable; residence in cities may enhance cognition because a city environment will reflect better the community development that has taken place in Taiwan after the Second World War [34] and such residence also tends to offer more opportunities for exposure to cognition-related stimuli. A dichotomous indicator was used for place of residence in 2006 and this was coded as 1 if the participant resided in a “city”, and as 0 if the participant resided in a “town”, namely lived outside of a small or large Taiwanese city.

The presence of at least one APOE4 allele was included. The analyses of APOE genotype used the polymerase chain reaction amplification refractory mutation system (PCR-ARMS) and polymerase chain reaction restriction fragment length polymorphism (PCR-RFLP) [25]. APOE4 was categorized in terms of whether or not participants carried at least one copy of the E4 allele (yes/no/unknown) [35].

Life stress factors salient to mental health include family living arrangements [21, 22], related family stress, and financial hardship [20, 36]. Family living arrangement can be used as a proxy for family care-giving and consisted of two dichotomous indicators, namely whether the subject was living with a partner and whether the subject was living with their grandchildren. The family stressor measure consisted of four items: stress/anxiety related to family relationships, stress/anxiety related to family health, stress/anxiety related to the family’s finances, and stress/anxiety related to work and the family. A dichotomous indicator was used for each item, namely 1 = “stressed” vs. 0 = “not stressed” and the four items were summed to produce a stressor scale that consisted of scores ranging from 0 to 4. A higher score is associated with greater stress within the family environment. The subjective experience of economic strain is more than just income and has been shown to be closely linked to emotional deprivation among older adults [19]. Economic strain thus was assessed by determining whether an individual and/or his/her spouse had enough living expenses and the responses ranged from 1 for “enough” to 4 for “much difficulty”.

As a life-related buffer factor, weekly exercise was assessed by self-reporting on the frequency of exercise each week, and this exercise was separated from any activity that improved the mind as well as the body such as chi kung, yoga, tai chi and others. Exercise was divided into three categories: less than once per week, two to five times per week, and more than six times per week [35]. The other covariates in this study consisted of the individual demographic characteristics of the participants (gender and ethnicity) [37] and whether the individual had depressive symptomatology [38, 39]. The ethnic groups used reflected the patterns of migration that have produced post-industrial Taiwan (Fukianese, Hakka, and Mainlander).

The presence of depressive symptomatology was measured using the Center for Epidemiologic Studies–Depression (CES-D) scale hat has a possible score range of 0–30 [40]. Higher scores represented higher levels of depressive symptoms. The ten CES-D items are listed in the Appendix. As suggested by Zhang et al. [35], individuals with a chronic disease were excluded from this analysis in order to reduce the endogeneity when the APOE gene and sociobehavioural factors are present as part of the analysis.

### Statistical analysis

The present study examined associations between mid-life SES, city residence, and general cognitive status separately for the Baby Boomer and pre-Boomer cohorts. The process started with bivariate analyses that characterized the sample distributions by birth cohort and gender. Next, multivariate regression models were conducted to examine the association between cognitive status and mid-life SES, city residence, and other independent variables that may affect cognitive status, such as the presence of an APOE4 allele and family care-giving. Due to the nature of the multistage cluster sampling used by the SEBAS survey, participants within the same community cluster may share some unobserved homogeneity and this might lead them to be correlated with each other. How cognitive status is clustered was examined at the primary sampling unit (PSU). The random effect at the PSU level were found to be significant (*p* < 0.05), and the intraclass correlation coefficient was 0.07. As suggested by Zhang and his colleagues [35], multilevel regression was thus employed in the present study.

To test whether mid-life SES and city residence are jointly associated with general cognitive status, an interaction term of mid-life SES with city residence was included. In this step and other analyses not shown here, the widowhood effect was tested for, as well as the interactions between mid-life SES and APOE4/gender. The aim was to determine whether the socioeconomic effects varied with the APOE4 and/or gender for each cohort. However, none of these effects added significantly to the models. All the analyses above were carried out separately for the Baby Boomers and the pre-Boomers. A final analysis was conducted with the data from both cohorts pooled together using a dummy variable to indicate the cohort difference. The interactions between the cohorts and the explanatory variables were tested to explore possible cohort differentials regarding cognitive status. In the interest of parsimony, any non-significant interactions were not included in the multivariate models.

STATA 13 [41] was used for data management and analysis. All of the above analyses were conducted using the maximum likelihood estimation method. The variables in the regression models may be highly correlated, thus an analysis of the variance inflation factor (VIF) was employed to assess multicollinearity. VIFs less than 5 were not regarded as of concern with respect to multicollinearity.

## Results

Table 1 presents the characteristics of the study participants across the two birth cohorts by gender. Compared to men, a higher percentage of women reported having a low SES, being widowed, living with grandchildren, and having higher levels of depressive symptoms. Compared to the pre-Boomer cohort, a greater portion of the Baby Boomer cohort reported having a high SES and that they were living with a partner.

**Table 1:** Distribution of demographic and socio-behavioral characteristics [mean (SD) or percentage] by gender and birth cohort, Taiwan, 2006

	Female			Male		
	Baby Boomers (born 1946–1964)	Pre-Boomers (born before 1946)	*p*-value	Baby Boomers (born 1946–1964)	Pre-Boomers (born before 1946)	*p*-value
	*N* = 275	*N* = 315		*N* = 257	*N* = 396	
Mid-life SES (%)			0.00			0.00
Low	0.00^b^	18.57		0.00^b^	10.63	
Medium	54.84	45.27		49.74	50.52	
High	45.16	36.16		50.26	38.85	
City residence in 2006 (%)	53.91	44.51	0.06	51.55	43.08	0.10
APOE4 (%)			0.00			0.00
Non-carrier	62.54	83.57		67.45	85.49	
Carrier	11.81	16.43		10.70	14.51	
Unknown	25.64	0.00^a^		21.85	0.00^a^	
Economic strain (%)			0.81			0.04
Enough	7.12	7.31		10.63	6.59	
Just enough	73.46	70.04		65.14	68.10	
Some difficulty	14.57	16.54		22.29	19.09	
Much difficulty	4.86	6.12		1.93	6.21	
Marital status (%)			0.00			0.00
Currently married	80.15	58.74		89.98	82.73	
Widowed	15.15	39.42		2.79	11.94	
Never married/separated/divorced	4.70	1.84		7.22	5.33	
Family living arrangement (%)						
Living with partners	77.11	54.15	0.00	89.85	79.35	0.00
Living with grandchildren	32.92	51.77	0.00	19.39	43.45	0.00
Family stressor (%)			0.21			0.04
0	55.96	60.91		61.39	65.00	
1	18.03	14.57		19.82	12.28	
2 and more	26.00	24.52		18.79	22.72	
Weekly exercise (%)			0.01			0.00
Less than 1	23.65	14.71		20.08	13.24	
2–5	52.80	59.15		60.72	53.78	
6+	23.55	26.15		19.19	32.98	
Depressive symptoms [mean (SD); range]	4.43 (5.59; 0–26)	6.04 (6.11; 0–26)	0.00	3.25 (4.65; 0–30)	4.68 (5.33; 0–28)	0.00
Age [mean (SD); range]	56.39 (2.12; 53–60)	70.29 (6.57; 61–85)	0.00	56.32 (2.14; 53–60)	70.88 (6.73; 61–85)	0.00
Ethnicity (%)			0.27			0.00
Fukianese	81.21	77.21		72.01	70.30	
Hakka	13.95	17.74		22.07	13.00	
Mainlander	4.84	5.06		5.92	16.69	
Cognitive score [mean (SD); range]	20.20 (3.87; 5–28)	16.51 (5.13; 0–26)	0.00	21.30 (3.20; 8–27)	18.45 (4.32; 0–27)	0.00

*N* is unweighted; percentages and means are weighted. Percentages may not sum to 100 owing to rounding. Chi-square tests were used for testing by birth cohort the significance of categorical variables and t-tests were used for testing by birth cohort the significance of continuous variables. ^a^Two observations were excluded due to missing APOE status. ^b^The five low SES individuals in this cohort were combined with the medium SES group

A multilevel model was used to examine the relationships being investigated separately among the Baby Boomers and pre-Boomers (Table 2). Among the Baby Boomers, older adults with a high mid-life SES were more likely than those with a low mid-life SES to have a significantly higher level of cognitive status (β = 2.81, *p* < 0.01). City residents had a higher level of cognitive status than town residents (β = 1.47, *p* < 0.01). In addition, a significant mid-life SES effect involving interaction with city residence was observed (β = −2.12, *p* < 0.01); this indicates that while city residents have higher levels of cognitive status in general, nevertheless town residents who are part of a social group with a high mid-life SES have an even higher level of cognition. Among stressor variables, a significant association was found between living with a partner and better cognitive status (β = 1.25, *p* < 0.01). Low levels of cognitive status were found to be associated with being female and being Fukianese.

**Table 2:** Multilevel regression estimates of the factors associated with cognitive function among Baby Boomers and pre-Boomers, Taiwan, 2006

	Baby Boomers (born 1946–1964)			Pre-Boomers (born before 1946)			All cohorts		
	Est.		SE	Est.		SE	Est.		SE
*Explanatory variables*									
Mid-life SES (ref = Low)									
Medium				1.98	**	0.51	2.07	**	0.45
High	2.81	**	0.50	3.56	**	0.55	3.61	**	0.47
Residence in 2006 (ref = town residence)									
City residence	1.47	**	0.49	−0.15		0.46	0.05		0.35
Mid-life SES-city residence interaction									
High mid-life SES × City residence	−2.12	**	0.62						
APOE4 allele (ref = non-carrier)									
Carrier	−0.44		0.45	−0.11		0.46	−0.16		0.33
Unknown	−0.15		0.34				−0.04		0.41
Economic strain (ref = Enough)									
Just enough	−0.48		0.49	−0.24		0.63	−0.28		0.41
Some difficulty	−0.67		0.61	−0.14		0.75	−0.32		0.50
Much difficulty	−1.19		0.96	−0.53		0.95	−0.62		0.68
Family living arrangement									
Living with partners (ref = No)	1.25	**	0.38	1.18	**	0.37	1.27	**	0.27
Living with grandchildren (ref = No)	−0.47		0.34	−1.25	**	0.33	−1.01	**	0.24
Family stressor (ref = 0)									
1	−0.54		0.37	0.32		0.50	−0.07		0.32
2 and more	0.49		0.37	0.88	*	0.42	0.78	**	0.29
Weekly exercise (ref = 2–5 times)									
less than 1 time	−0.54		0.35	−1.72	**	0.48	−1.09	**	0.31
6+ times	−0.35		0.41	−0.85		0.52	−0.46		0.34
Depressive symptoms	−0.03		0.03	−0.16	**	0.03	−0.04		0.04
Female gender (ref = Male)	−0.75	*	0.29	−1.17	**	0.35	−0.95		0.23
Ethnicity (ref = Fukianese)									
Hakka	1.03	*	0.42	0.86		0.56	0.74	*	0.37
Mainlander	1.30	*	0.60	−0.73		0.48	1.26		0.72
*Cohort comparison*									
Pre-Boomers (ref = Boomers)							−1.73	**	0.33
Pre-Boomers × Depressive symptoms							−0.12	**	0.04
Pre-Boomers × Mainlander							−1.97	*	0.82

^*^ *p* < 0.05, ^**^ *p* < 0.01

Among pre-Boomers, a significant and positive association was shown between mid-life SES and cognitive status. Compared to older adults with a low mid-life SES, older adults with either a medium or high mid-life SES were found to have significantly better cognitive status (β = 1.98, *p* < 0.01 and β = 3.56, *p* < 0.01, respectively). When the other stressor variables were taken into account, the results demonstrated that living with a partner was associated with a higher level of cognitive status for this cohort (β = 1.18, *p* < 0.01), but that living with grandchildren was associated with a lower level of cognition for this cohort (β = −1.25, *p* < 0.01). A lower level of general cognitive status was further found to be associated with being female (β = −1.17, *p* < 0.01) and having depressive symptomatology (β = −0.16, *p* < 0.01). Contrary to expectations, a significant and positive association between the number of family stressors and general cognitive status was observed (β = 0.88, *p* < 0.01).

In the model with all cohorts, the findings for the main cohort effect and its interactive effects with depressive symptoms and ethnicity indicated that the pre-Boomer cohort has lower levels of cognitive status (β = −1.73, *p* < 0.01) and, in particular, the lower levels of cognition were associated with depressive symptomatology (β = −0.12, *p* < 0.01) and being a Mainlanders (β = −1.97, *p* < 0.01) among these pre-Boomers.

## Discussion

The present study yields several important findings. First, the results appear to support the relationship between social disparities and cognitive status [7, 8] by showing a significant association between mid-life SES and cognition among both Baby Boomers and pre-Boomers and this relationship persisted even after taking genetic and stressor factors into consideration. Second, the results are consistent with the hypothesis that there is an interaction between mid-life SES and city residence. However, the analyses demonstrate that this interaction effect occurs only among the Baby Boomers but not among the pre-Boomer cohort; in this context it should be noted that the changing socioeconomic context of Baby Boomers has led them to be categorized as one of the most distinct social groups in Taiwan [10, 11]. Finally, consistent with prior research, cognitive status was found to be lower among elderly family caregivers [20] and was found to be higher among older people who undertake weekly exercise [23, 24].

The results from the present study substantiate previous studies that indicate an adverse effect of SES disadvantage on cognitive status [1, 7–9, 32, 37, 41]. The findings extend such results by showing a significant effect of mid-life SES on general cognitive status in later life among older adults with a high SES compared to those who with a low or medium SES for both cohorts, even controlling for the presence of the APOE4 allele, life stress and a wide range of individual characteristics. As suggested by the exposure-disease-stress model, people with a lower SES have limited socioeconomic resources and this in turn is associated with poorer long-term health [6]. Mid-life SES was measured by combining education attainment of the participants and spouses and the major mid-life occupation of the participants and spouses. Such mid-life SES indicators may warrant a need of household-level strategies and policies that target social disparities and cognitive health in Asian societies.

Another important finding of the present study is empirically to demonstrate a significant interaction effect between mid-life SES and city residence among Baby Boomers and this effect is absent among pre-Boomer cohort. This may be partly due to the fact that a Baby Boomer’s mid-life SES represents not only their personal abilities but also their social expectations. Baby Boomers are much more likely than pre-Boomers during their mid-career to have been involved in a struggle between their work and family roles, particularly because job opportunities are more concentrated in cities than towns after the rapid urbanization of Taiwan in the 1980s. In summary, the findings of this study are in agreement with the exposure-disease-stress model that suggests the importance of residence of place as an important covariate when trying to understand effect of mid-life SES on Baby Boomers [6]. Future studies on this issue are needed and they should be both residence sensitive and cohort appropriate.

The present study has produced significant evidence with regard to cohort differences in cognitive status. Consistent with prior research, there is a significant effect whereby living with a partner is associated with better cognition [1]. This beneficial effect is present among both Baby Boomers and pre-Boomers. Interestingly, the analyses indicated that there is a negative effect of living with grandchildren, but only among pre-Boomers. One explanation for this inverse association between living with grandchildren and cognitive status is that, because ageing pre-Boomers have poorer cognitive status than Baby Boomers, there is a need for the family to care for these pre-Boomers and thus the pre-Boomers need to live with their children and, by association, with any grandchildren. Another possible interpretation is that caregiving for grandchildren is exhausting; such exhaustion may act as a precursor to a feeling of uselessness that in turn may be associated with poorer cognition [42]. In addition, ethnic differences in cognitive status were found and Fukianese pre-Boomers have poorer cognition than Mainlander Baby Boomers. This may be partly due to the fact that being Fukianese represents not only a personal ethnicity but also has involved sociopolitical forces in recent times that have elicited various ethnic disparities such as stigma and discrimination. These will have had consequential effects on this subgroup.

Unexpectedly, in the presence of the APOE4 allele, perceived economic strain and family stress were associated with lower levels of cognitive status only when the bivariate analyses were applied. As stressors have been speculated to cause increased neuron loss via the glucocorticoid pathways [6, 7, 43], this finding seems to suggest complex gene-environment interactions must have an effect on how cognitive status develops with age. More research is needed to explore the implications of this social etiology and even to investigate how the cohorts differ in terms of their cognitive status in this context.

The use of multilevel modeling that fits complex sampling strategies in this study avoids possible estimation bias. Moreover, this research innovatively detected cohort differences, mid-life SES, and an interaction effect between mid-life SES and city residence all of which were acting on cognitive status simultaneously. Nevertheless, this work is not without its limitations. First, the study excluded non-self-respondents (*n* = 39) because the cognitive outcome is based on self-reporting and thus cannot be assessed via proxy. This exclusion involved less than 3% of the sample and thus should have only a minor effect on the results. Second, the sample included 24% of Baby Boomers who had an unknown APOE status. If the subgroup with unknown APOE status was excluded, this would significantly decrease the sample size and consequently the statistical power of the multivariate analyses. However, in the present study, APOE status was found not to be significantly associated with cognitive function among either Baby Boomers or pre-Boomers. It was possible to further assess the differences in individual socioeconomic status and physical health between the known and unknown groups of APOE for the Baby Boomer cohort (the results not tabled). The analyses indicated that there was no significant difference in terms of APOE status. As a result, it seems unlikely that the findings are not seriously biased by inclusion of the unknown AOPE status individuals. Third, the SEBAS dataset is based on the self-reported recall of mid-life SES and stressor variables, which raises the issue of recall bias among older pre-Boomers. Fourth, while prior research has suggested the importance of residential characteristics in relation to cohort differences in cognitive status (namely neighborhood SES characteristics and residential stability), such information was not available to the present investigation. Fifth, the scope of this study was limited to comparing general cognitive status between Baby Boomers and pre-Boomers and thus is designed to underscore the significant impacts of the rapid social changes that occurred after World War II in Taiwan. As both cohort groups comprise multiple birth years, future research may need to become more specific in terms of each birth year because these contextual changes varied greatly over the time period studied, especially in terms of the pre and post 1945 Taiwan. Specifically, there have been particular changes in Taiwan that did not occur in Europe and the US. Lastly, any cross-sectional data analysis cannot disentangle or establish the causal links suggested herein. However, this theory-based study using the multilevel analytical approach does provide important insights and identifies independent effects related to mid-life SES that have affected Baby Boomers and pre-Boomers in Taiwan.

## Conclusions

Despite the above limitations, to our knowledge there has been little research on Asian populations that has used survey data with genetic information in order to explore cohort differences related to the association between mid-life SES, city/town residence, and cognitive status among community-based older adults. The findings of the present study demonstrate an association between cohort differences and general cognitive status, which further highlights the fact that cognitive function is shaped by the birth cohort of an individual in relation to their socioeconomic context and residential place. This study also provides evidence that individuals with better cognitive ability are more likely to live with a partner and to have weekly exercise. These findings are of crucial importance to world populations because older people are increasingly becoming burdened with cognitive health problems [1]. Policy-makers should take the cohort differences associated with mid-life SES and the related inequitable distribution of socioeconomic resources into consideration and begin developing programs and interventions that are aimed at promoting in a targeted manner cognitive health and healthy aging among specifically defined cohorts.

## Appendix: The 10 CES-D items

Everyone has mood changes. In the past week, have you experienced the following situations or feelings? If yes, continue to ask: Does this happen to you rarely (one day in the past week), sometimes (2–3 days in the past week), often or chronically (over 4 days in the past week)?

1. Not interested in eating, have a poor appetite.
2. Feel that doing everything was exhausting.
3. Sleep poorly (unable to sleep soundly).
4. Feel you were in a terrible mood.
5. Feel lonely (isolated, with no companion)
6. Feel people around you weren’t nice to you (unfriendly).
7. Feel anguish.
8. Unable to gather your energy to do things (Had no will to do anything).
9. Feel joyful.
10. Feel that your life was going well.

## Abbreviations

APOE4: Apolipoprotein E4
CES-D: Center for epidemiologic studies–Depression
PSU: Primary sampling unit
SEBAS: Social environment and biomarkers of aging study
SES: Socioeconomic status
TLSA: Taiwan longitudinal survey of ageing
